# Identification and Characterization of the Intra-Articular Microbiome in the Osteoarthritic Knee

**DOI:** 10.3390/ijms21228618

**Published:** 2020-11-16

**Authors:** Joseph C. Tsai, Grant Casteneda, Abby Lee, Kypros Dereschuk, Wei Tse Li, Jaideep Chakladar, Alecio F. Lombardi, Weg M. Ongkeko, Eric Y. Chang

**Affiliations:** 1 Department of Surgery, Division of Otolaryngology-Head and Neck Surgery, UC San Diego School of Medicine, San Diego, CA 92093, USA; jctsai@ucsd.edu (J.C.T.); gecastan@ucsd.edu (G.C.); acl008@ucsd.edu (A.L.); kderesch@ucsd.edu (K.D.); wtl008@ucsd.edu (W.T.L.); jchaklad@ucsd.edu (J.C.); rongkeko@health.ucsd.edu (W.M.O.); 2 Research Service, VA San Diego Healthcare System, San Diego, CA 92093, USA; alecio.lombardi@icloud.com; 3 Department of Radiology, University of California, San Diego, CA 92093, USA

**Keywords:** microbiome, bacteria, knee, osteoarthritis, synovium, inflammation

## Abstract

Osteoarthritis (OA) is the most common joint disorder in the United States, and the gut microbiome has recently emerged as a potential etiologic factor in OA development. Recent studies have shown that a microbiome is present at joint synovia. Therefore, we aimed to characterize the intra-articular microbiome within osteoarthritic synovia and to illustrate its role in OA disease progression. RNA-sequencing data from OA patient synovial tissue was aligned to a library of microbial reference genomes to identify microbial reads indicative of microbial abundance. Microbial abundance data of OA and normal samples was compared to identify differentially abundant microbes. We computationally explored the correlation of differentially abundant microbes to immunological gene signatures, immune signaling pathways, and immune cell infiltration. We found that microbes correlated to OA are related to dysregulation of two main functional pathways: increased inflammation-induced extracellular matrix remodeling and decreased cell signaling pathways crucial for joint and immune function. We also confirmed that the differentially abundant and biologically relevant microbes we had identified were not contaminants. Collectively, our findings contribute to the understanding of the human microbiome, well-known OA risk factors, and the role microbes play in OA pathogenesis. In conclusion, we present previously undiscovered microbes implicated in the OA disease progression that may be useful for future treatment purposes.

## 1. Introduction

Osteoarthritis (OA) is one of the most common and well-documented diseases, with its burden on humanity dating back to prehistoric times [1]. It is the most common form of arthritis and is one of the leading sources of pain and disability in the elderly [2,3]. Despite its debilitating effects on one’s quality of life, there remains no cure for OA. Current treatment options are limited to symptomatic interventions [4,5], such as exercise and non-steroidal anti-inflammatory drugs, which have no impact on disease progression [6,7]. With life expectancies and obesity rates set to increase worldwide [8,9], research into OA pathogenesis grows increasingly relevant. While many studies have clearly correlated chronic low-grade inflammation to cartilage degeneration, the exact mechanisms for the pathogenesis of OA are still poorly understood [10,11].

The human gut microbiome has recently been shown to have both direct and indirect impacts on host physiology, including metabolic and immune functions [11]. Changes in the gut microbiome have been implicated in numerous diseases, especially those in which chronic inflammation is central to disease severity and progression. These include allergic asthma [12], rheumatoid arthritis [13] and osteoporosis [14]. Additionally, age has been identified as a top risk factor for OA and has been associated with chronic inflammation [15]. Since the gut microbiome composition has also been shown to change with age [16] and intestinal permeability increases with age [17], it may not be surprising if harmful microbes “leak” into surrounding tissues and contribute to chronic, age-related inflammation [18]. The pro-inflammatory phenotype caused by microbial dysbiosis can lead to the improper surveillance of microbial communities, resulting in OA [19].

Recent research suggests that the migration of pathogenic bacteria from the gut to the synovium in OA patients can contribute to the pathogenesis of OA [20,21]. While joints were initially thought to be sterile, mounting evidence points to a synovial fluid and synovial tissue microbiome [22,23]. Unlike the heavily researched gut microbiome, there has been scarce research performed on this new intra-articular microbiome, especially in OA. The interaction between intra-articular microbes in OA and immune-associated signaling pathways has not been characterized. Therefore, in this study, we aim to identify and characterize the diversity and composition of the microbiome in the synovium of OA patients to unveil its relevance to key immunological and metabolic signatures. In doing so, we hope to better understand the role of the synovial microbiome in modulating immune activity and altering cell signaling pathways, offering a clearer picture of OA pathogenesis.

## 2. Results

### 2.1. Differential Microbial Abundance in OA and Normal Tissue

**Figure 1:**
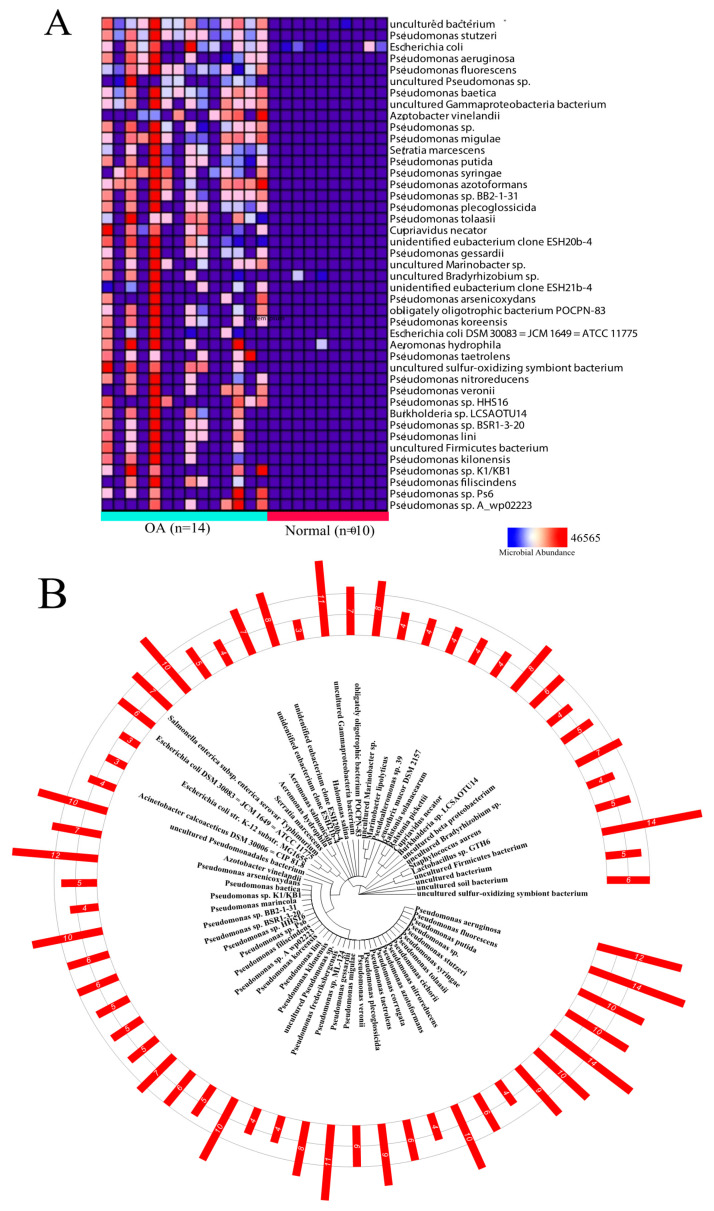
Summary of differential presence analysis. (**A**) Heatmap visualizing the differentially present microbes in OA vs. normal samples. (**B**) Phylogeny tree of differentially present microbes with red bars representing the number of OA patients with a microbial presence for each microbe.

PathoScope 2.0 [24] was used to align patient sequencing data to bacterial reference genomes, outputting total bacterial abundance based on read count data. In total, 299 bacterial species were identified from the OA samples and 84 bacterial species were identified from the normal samples. Not all 84 species identified from the normal samples were found to be present in the OA samples. In fact, only 36 species overlapped. Microbial abundance data was compared between OA and normal samples to identify differentially abundant microbes. Fourty-three microbes were identified to be significantly dysregulated between the two patient cohorts (logFC > |2|) (Figure 1A). Of all differentially abundant microbes, almost half were *Pseudomonas* (Figure 1B). We note that there is one OA sample that outstands from other samples with a significantly high abundance, indicated by the consistent red boxes in an entire column in Figure 1A. While there are usually outliers in cohorts for reasons that are difficult to pinpoint, we can suspect the reasons causing this increase in microbial abundance to include diet, diabetes, or other metabolic disorders, all of which can induce an increase in gut permeability, and consequently an increase in intra-articular microbial abundance.

### 2.2. Microbes Are Significantly Correlated with Catabolic and Anabolic Pathways

We used the Gene Set Enrichment Analysis (GSEA) and the Weighted Gene Co-expression Network Analysis (WGCNA) to correlate the abundance of microbes of interest to the expression of genes in immune signaling pathways and signatures. Out of all dysregulated microbes, nine were found to be significantly correlated to immune signatures and OA pathways including *Pseudomonas koreensis*, *Pseudomonas pessardii*, *Pseudomonas mingulae*, *Pseudomonas arsenicoxydans*, *Pseudomonas azotoformans*, obligately oligotrophic bacterium *POCPN-83*, unidentified eubacterium clone *ESH20b-4*, uncultured bacterium (77133), and *Cupriavidus necator*.

**Figure 2:**
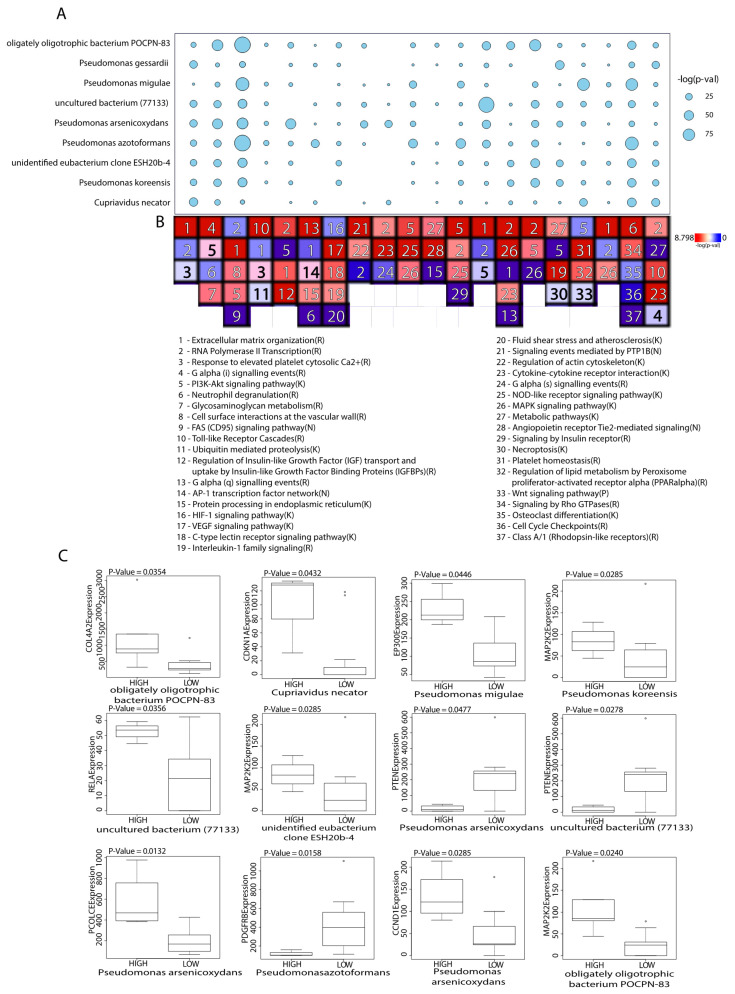
Pathways and genes associated with GSEA-correlated microbes. (**A**) Balloon plot illustrating the microbes and the strength of their association to gene clusters using weighted correlation network analysis (WGCNA). The significance of the correlation is represented by the size of the balloon, where the larger the balloon, the more significant the correlation. (**B**) Heatmap showing the top pathways of each cluster of genes found using Reactome Fi. (**C**) Boxplots presenting the association for the most significant IA genes involved within the top pathways.

Figure 2 shows the results of WGCNA, which show significantly enriched or depleted pathways implicated in OA, confirming the pathways we found to be significantly correlated to microbes in GSEA. In both our GSEA and WGCNA results, we found most pathways either upregulated because of involvement with inflammation-induced extracellular matrix (ECM) remodeling or downregulated because of increased ECM catabolism. For example, while upregulation of ECM organization pathways and interleukin signaling pathways was observed in numerous *Pseudomonas*, we also found the glycosaminoglycan degradation pathway to be downregulated in the unidentified eubacterium clone *ESH20b-4*. Interestingly, many upregulated pathways involved in the synthesis of collagen and general ECM signaling interactions were significantly correlated to increased abundance of the uncultured bacterium (77133).

Boxplots showing the associations between the immune-associated (IA) genes of the top pathways and the 9 GSEA-correlated microbes are displayed in Figure 2C. Of the nine significantly correlated IA genes, most have previously been involved in osteoarthritis pathogenesis or have a role in inflammation. For instance, a positive relationship has been identified between *RELA* expression and *MMP-13* expression, a cytokine involved in cartilage degradation, in synoviocytes affected by osteoarthritis [25]. *EP300* has been shown to repress autophagy [26], which plays an important role in protecting chondrocytes from oxidative stress and is constitutively expressed in normal cartilage [27]. *CDKN1A* expression levels have been associated with senescent cells, which play a role in OA pathogenesis [28]. *CCND1* and ECM-related *PCOLCE* are upregulated in OA cartilage tissues [29,30]. *PTEN,* which was negatively correlated with uncultured bacterium (77133) and *Pseudomonas arsenicoxydans*, inhibits Akt signaling that triggers chondrocyte senescence [31]. *PDGFRB* is critical for vasculogenesis and in OA has been shown to drive pathological subchondral bone angiogenesis [32].

While uncultured bacterium (77133) was observed to be involved in the upregulation of most of the ECM signaling and collagen pathways, the other microbes were significantly correlated to the downregulation of other signaling pathways crucial to joint and immune function. Wnt and Rho GTPase signaling pathways have a synergistic role in transducing signals from the ECM to govern numerous aspects of cell physiology and were both found to be downregulated [33]. In addition, the neutrophil degranulation pathway was also downregulated. This OA-preventive pathway secretes antimicrobial cytotoxins and acts to defend the body against microorganisms, and its downregulation in the OA samples may explain the increased microbial abundance over the normal samples [34].

**Figure 3:**
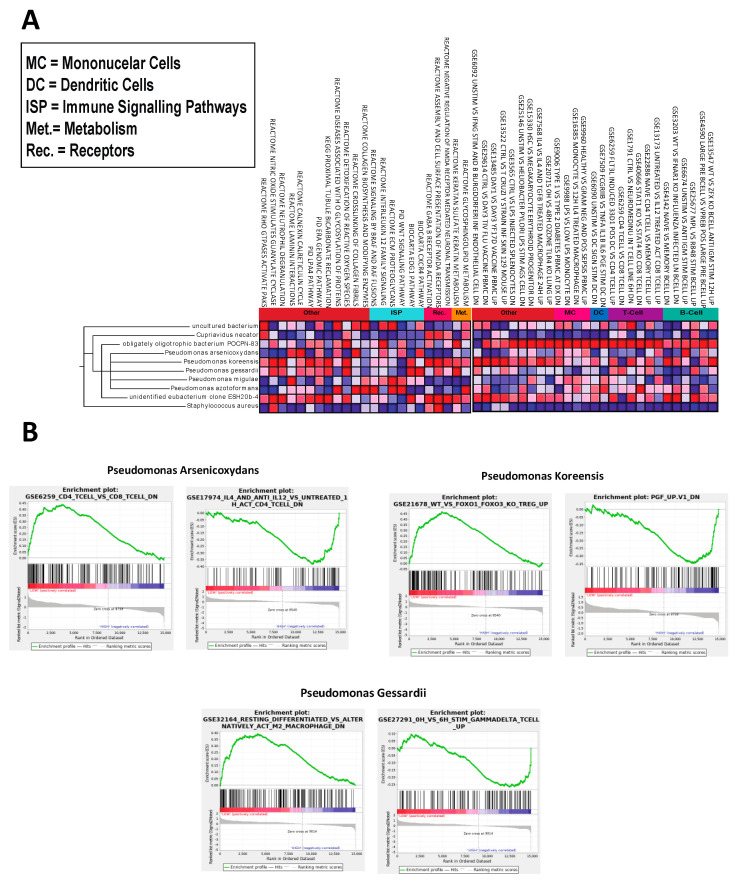
(**A**) Gene set enrichment analysis (GSEA) correlations of significantly upregulated microbes including multiple *Pseudomonas*, Unidentified Eubacterium Clone *ESH20b-4*, Obligately Oligotrophic Bacterium *POCPN-83*, Uncultured Bacterium (77133), and *Cupriavidus Necator* with canonical pathways and immunologic signatures (nominal *p* < 0.05). (**B**) Select GSEA plots of immunogenic signatures for the top three most significant microbes. A peak on the left side of the plot indicates that higher abundance of the microbe correlates with higher expression of the genes in the gene set whereas a valley on the right side of the plot indicates that lower abundance of the microbe correlates to lower expression of the genes in the gene set.

All canonical pathways significantly correlated to the nine key microbes can be found in Figure 3A. The raw GSEA plots of the top correlated pathway of the six other key microbes not shown in Figure 3B can be found in Supplementary Figure S1. Bar graphs depicting all significant pathways and immune signatures correlated with the nine key microbes can be found in Supplementary Figures S2 and S3.

### 2.3. Microbes Are Significantly Correlated with OA Immunologic Gene Signatures

An advantage of GSEA over WGCNA is its unique ability to interpret common perturbations in clusters of genes to identify gene signatures. Most immunologic gene signatures correlated to microbes are of cells in both the innate and adaptive immune systems, such as macrophages, mast cells, T-cells, B-cells, and monocytes (Figure 3B). The dysregulation of gene signatures correlated to each microbe can be visualized in Figure 3A.

### 2.4. Microbes Are Significantly Correlated with Immune Cell Types

**Figure 4:**
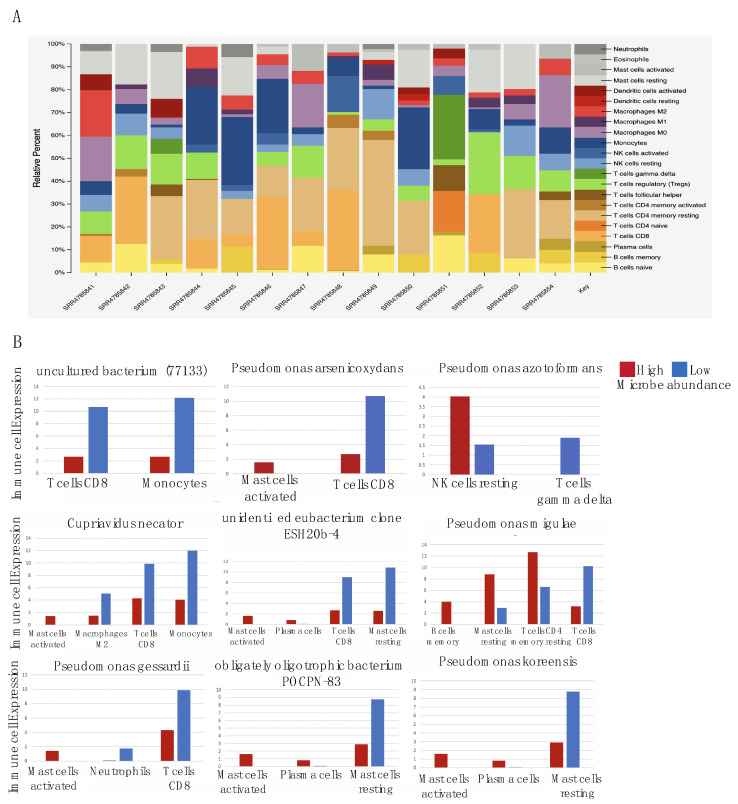
(**A**) Relative expression of different immune cell types amongst OA patients. (**B**) Boxplots presenting the association between abundance of 9 GSEA-correlated microbes and different immune cell types.

The relative expression of different immune cell types in OA patients are illustrated in Figure 4A. We also found that the binarized abundance of seven GSEA pathway-associated microbes were significantly correlated with at least one immune cell type (*p* < 0.05) and two were significantly correlated with at least one immune cell type (*p* < 0.1) (Figure 4B). The cutoff for statistical significance was increased to 0.1 for the last two microbes to incorporate all nine of our key pathway-correlated microbes. Six out of the nine microbes (*Cupriavidus necator,* unidentified eubacterium clone ESH20b-4, *Pseudomonas gessardii, Pseudomonas koreensis,* obligately oligotrophic bacterium *POCPN-83, Pseudomonas arsenicoxydans)* were positively correlated with activated mast cells infiltration. We also found that another set of six microbes were negatively correlated with CD8+ T cells.

### 2.5. Negligible Contaminants Found in Differentially Abundant Microbes

With the advent of new high-throughput sequencing technologies, trillions of nucleotide bases are able to be recognized, allowing for profiles of microbial communities to be obtained [35]. However, as a result of this powerful sequencing technology, the potential of also returning sequencing derived from environmental contamination such as laboratory reagents or handling of tissue samples by hospital workers is introduced [36]. To distinguish between nascent microbial reads and contaminant reads in our samples, we employed Spearman’s correlation to compare individual microbe abundance levels to total microbial reads in each patient. A microbe was deemed nascent to the biopsy sample if the regression line was a slope. Contrastingly, if there was a nearly vertical line, the microbe was considered to be of contamination. This classification is based on the idea that contaminant microbes would yield a similar microbial abundance in all samples, regardless of the size of the tissue. Since the size of this tissue is represented by total microbial abundance, as the reads of an individual microbe increases, the total microbial abundance should also increase proportionally. After conducting this contamination correction analysis on all OA and normal samples, we found no microbes with a nearly vertical regression line, even after accounting for outliers. Although we can confidently claim the authenticity of the nine GSEA-correlated microbes because of the strong linear association, we were unable to confirm the biological relevance of many other dysregulated microbes with this method of contamination correction.

**Figure 5:**
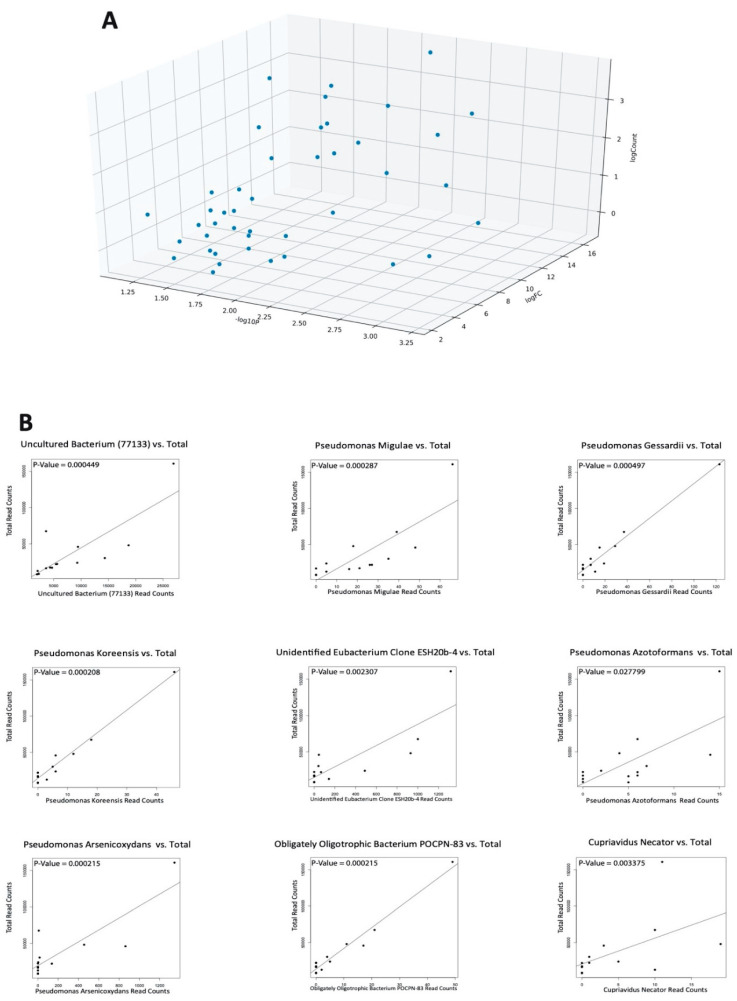

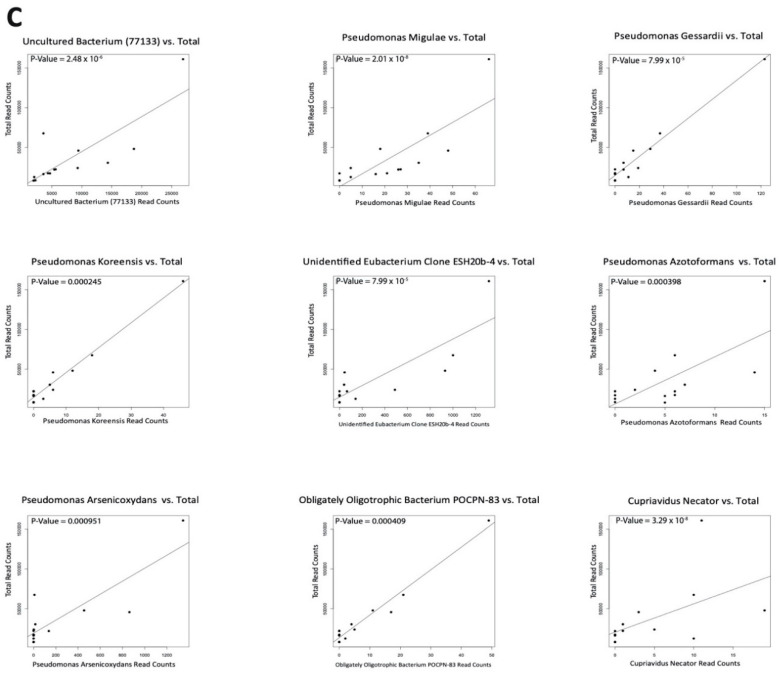
Contamination correction screening using Spearman’s correlation for GSEA-correlated microbes. All microbes with a positive slope of total microbial read count vs. read count of the specific microbe are shown here. (**A**) Differentially abundant microbes from comparing OA samples vs. normal samples (Kruskal–Wallis Test, *p* < 0.05). (**B**) Scatter plots of normal samples. (**C**) Scatter plots of OA samples.

However, we found that most significant microbes were not potential contaminants because of their high fold change values (Figure 5A). Since all OA and normal samples were sequenced by Guo et al., as well as the fact that both the OA and normal synovial biopsies were obtained from the same surgical facility, it is logical to assume that the handling and sequencing of tissues follow the same procedure [37]. As a result, we reached the conclusion that if a microbe is an environmental contaminant, its fold change would be similar between OA and normal samples. This is clearly false in our analysis as we found all dysregulated microbes to be dramatically different in abundance as seen in Figure 1A and further seen in the fold change values of Figure 5A. The plots with a strong linear association, indicating their biological relevance, are shown in Figure 5B. All other plots for the rest of the differentially expressed microbes can be found in the Supplementary Figure S4.

In summary, we did not observe extensive overlap in the bacterial groups in any of the GSEA-correlated microbes, nor any of the microbes that were significant through differential abundance analysis. While there is a chance that some skin or reagent contaminants were sequenced, none of the biologically relevant microbes were identified as contaminants and are likely true positives present within the synovium samples.

## 3. Discussion

In recent years, there has been an increasing number of studies linking the gut microbiome to OA onset [5,38,39,40]. Currently, amongst synovial joints, microbial communities have only been identified in the knee and hip. While how such microbes and their DNA reaches these body sites remains unclear, many have hypothesized that bacteria in the lumen can enter the blood stream when the permeability of the intestinal epithelial lining is compromised. Several factors have been proposed to contribute to a “leaky” gut. These include an individual’s diet, which changes the gut microbiota composition, affecting intestinal barrier function and subsequently leading to microbial translocation [41]. In addition, burn injuries and alcohol consumption can lead to increased gut permeability [42,43,44]. However, research on the precise role of the intra-articular microbiome in OA development is still largely underexplored. One of the primary challenges in studying sites aside from the gut is the relatively small number of microbes, which may amplify potentially erroneous computations due to the potential contamination of environmental bacteria when sequencing. Nevertheless, in our study, we found that we were able to identify legitimate, individual bacterial sequences from high-throughput RNA sequencing. This conclusion can be explained by the fact that all differentially abundant microbes were significantly more abundant in OA vs. normal, despite all biopsies being derived from the same medical facility and sequenced by the same sequencing center and research group. Furthermore, the variation in microbe abundances for a single microbe due to the difference in each biopsy sample shown in our Spearman correlation plots also supports the conclusion of negligible contamination reads.

After correcting for contamination, we identified 43 microbes that are differentially abundant between the OA patient cohort and the normal samples. These microbes were correlated to immunologic signatures and pathways using GSEA. Nine of these microbes were shown to have significant correlations to the tested pathways and signatures. To confirm the association of microbes to certain gene sets, we correlated each key microbe to individual genes and utilized WGCNA to cluster the correlations into specific pathways. Almost all pathways critical to the development of OA overlapped in our correlations of both methods. In addition, the abundance of these nine microbes has not yet been shown to be affected by NSAID consumption. We believe it is likely that these patients received NSAIDs prior to undergoing surgery, as they are widely accepted as first-line therapy for OA patients [45,46]. However, current research has shown that NSAIDs are primarily associated with four microbial groups: *Prevotella* species, *Bacteroids* species, family *Ruminococcacea* and *Barnesiella* species [47].

We demonstrate significant associations between many intra-articular bacteria species and critical processes associated with OA. From our results using individual microbes’ abundance, we can summarize the functions that dysregulated microbes affect in two ways: they play a role in increased inflammation-induced extracellular matrix remodeling, and they are involved in decreased cell signaling pathways crucial for joint and immune function. While all key microbes are involved in either both or at least one of these processes, some downregulate neutrophil degranulation to block against antimicrobial defenses of the immune system. In addition, important cell-governing signaling pathways such as the Wnt and Rho GTPase signaling pathways are also significantly downregulated, further explaining the increased catabolic activity. We also found that these microbes were correlated with immune cell expression. This is consistent with our findings that these microbes are correlated with the dysregulation of immunologic gene signatures, thereby increasing inflammation and contributing to OA pathogenesis, as current research shows that mast cells directly promote inflammation and cartilage damage in mouse models of osteoarthritis [48]. However, low microbe abundance is associated with high CD8+ T cell expression, which is unexpected because CD8+ T cells are known to worsen OA [49]. Instead, we believe these results could be due to other factors, such as the ratio of CD4+:CD8+ T cells. Research has found that the ratio of CD4+ to CD8+ T cells is higher in OA synovial tissue compared to that of normal synovial tissue [50], suggesting that increased microbe abundance is associated with a more skewed CD4+:CD8+ T cell ratio. However, further research must be done to confirm this.

Out of the nine key microbes significantly correlated to OA and immune pathways, five were *Pseudomonas*. While never identified in OA, *Pseudomonas* have previously been identified in the synovial fluid of patients with rheumatoid arthritis [51,52], as well as in patients with undifferentiated arthritis [53].

It is worth noting that the different microbes identified in this study compared to other published works are likely due to the microbiome being highly dependent on geography [54]. As our study is based on samples from Australia [37], many groups have also shown *Pseudomonas* to be a part of the microbiome of the Australian population. Ozkan and colleagues found *Pseudomonas* to be the most consistently detected genus in the ocular microbiome of Australian patients undergoing pterygium surgery [55], while Smith and colleagues found *Pseudomonas* in sputum samples of Australian patients with cystic fibrosis as the most dominant genus [56]. Furthermore, Dinsdale and colleagues found *Pseudomonas* to be even more enriched in the genital microbiome of aboriginal Australian women compared to that of non-aboriginal Australian women, further lending evidence to the unique link of *Pseudomonas* to the microbiome of the Australian population [57].

Our study has several limitations. Firstly, our study uses a small sample size of 14 OA and 10 healthy synovium samples. While we had sufficient patients to identify numerous dysregulated microbes, we could include more patients in future studies to identify additional microbes, as well as increase the reliability and statistical power of our results. Secondly, our study is limited to patients living in Australia. Given that the human microbiome is associated with geographical location and patient ethnicity [53], our future research could include patients of different ethnicities from different locations. Lastly, while we lacked explicit contamination “correction” for the entirety of microbes identified, we are confident that the key microbes we present are truly biologically relevant considering the large fold change and the fact that all samples, normal and OA, are sequenced at the same hospital and by the same research group.

In conclusion, we identified a distinct synovial microbiome in OA of Australian patients and, for the first time, link these microbes to key immunological and metabolic pathways known to play a role in the disease. Our results indicate that future studies of the intra-articular microbiome should be performed from different geographical locations in order to comprehensively characterize the various microbial compositions that are involved in the initiation and progression of OA. In addition, in light of a report by Dunn et al. that identified a microbial DNA signature in human knee and hip cartilage [58], future work could include assessment of the synergistic role of the cartilage and synovial microbiomes in OA development. Lastly, the concurrent assessment of the gut, blood and intra-articular microbiome would support the hypothesis that a “leaky” gut allows for microbial migration from the gut to the joint, contributing to OA pathogenesis.

## 4. Materials and Methods

**Figure 6:**
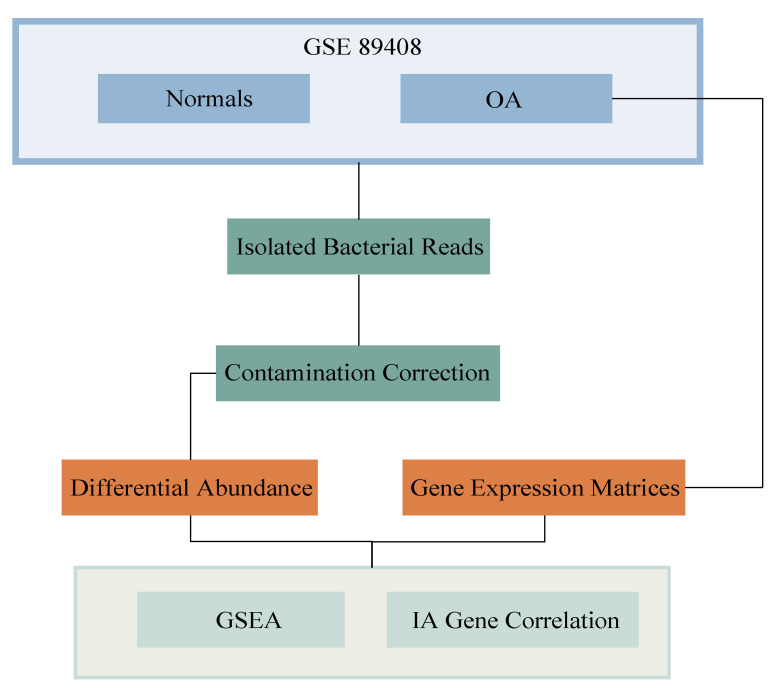
Schematic of analysis and workflow.

An overview of our methods is shown in Figure 6.

### 4.1. Acquisition of Data and Extraction of Isolated Bacterial Reads

RNA-seq of 14 OA and 10 healthy synovial biopsy samples were downloaded from GEO (accession code GSE89408; https://www.ncbi.nlm.nih.gov/geo/accessed 17 October 2019). Both cohorts of synovial biopsy samples were sequenced by Guo et al., and were obtained from a group of Australian patients. Healthy subjects were defined as those who had no evidence of any form of arthritis on history or examination and had no cartilage damage or synovitis. OA subjects had knee pain and following arthroscopic surgery, had supporting findings. Furthermore, these OA subjects already had a clinical history and/or examination finding suggestive of OA [37]. The average age of healthy donors was 37.8 y (range, 22–63 y). There were three females (30%) and seven males (70%). The average age of OA donors was 50.2 y (range, 19–69 y). There were eight females (57.1%) and six males (42.9%). As the risk of developing OA increases with age and women are more likely to develop OA than men, especially after 50, the biological variables for our dataset are quite representative of the average population [59]. We also conducted a multivariate logistic regression analysis to legitimatize our differential abundance data to take into account age and gender. We found that after adjusting for these variables, not only did all nine of our key biologically relevant microbes remain significant, the *p*-value of the differential abundance remained <0.00001.

PathoScope 2.0 was used to separate the microbe-specific reads incorporated in the human reads of high-throughput RNA-seq and align it to the reads in the target reference library, producing levels of microbe abundance and individual taxonomic lineage [24]. This direct alignment was done through Bowtie2, aligning the RNA-sequencing reads to both human and bacterial target reference libraries, both of which are comprised of genomes deposited in the National Center for Biotechnology Information (NCBI) nucleotides database (https://www.ncbi.nlm.nih.gov/nucleotide/). PathoScope 2.0 first identifies any reads in the transcriptome that correspond to the human genomic sequences. Reads that do not map to the human sequences are subsequently matched to the genomes of bacteria. PathoScope then generates two output measures quantifying the amount of bacterial species present in samples. One measure, best guess, quantifies the relative abundance of each species, expressed as a percentage. The other measure, best hit, signifies the absolute integer count of each species in the sequencing data. The best hit count data was used.

### 4.2. Differential Abundance

To compare the variations in joint microbiota profiles between normal and OA samples, the Kruskal–Wallis test was used to determine the dysregulated microbial abundance in the OA samples. All microbes with *p*-value < 0.05 and fold change >2 or <−2 were deemed differential in microbial abundance. Since microbial data is compositional, only microbes with a non-zero read count in more than four of the samples were significantly differentially abundant to account for the overabundance of zeroes in the data.

### 4.3. Acquisition of RNA Read Counts

First, FastQC was used to perform quality control checks on the raw sequence data [60]. After ensuring the quality of the data, STAR aligner was used to map the RNA-seq reads to produce files in the bam format [61]. These bam files were aligned sequences in compressed binary representation and required further manipulation to get the read counts. The Python framework Htseq-count was then used to convert bam into count [60]. These counts were then normalized into counts per million (CPM) for our subsequent analyses.

### 4.4. Gene Set Enrichment Analysis (GSEA)

GSEA was utilized to correlate the dysregulated microbes to gene sets including canonical pathways (C2:CP) and immunologic signatures (C7). The canonical pathways were compiled from various databases including Biocarta, Reactome, and the Kyoto Encyclopedia of Genes and Genotype (KEGG). The immunologic signatures were generated through the manual curation of previous literature regarding human and murine immunology. Using these gene sets, GSEA quantifies the synergistic gene regulation with an enrichment score and ranks the gene sets correlated to each microbe [62]. Canonical biologic pathways (C2:CP gene sets) and oncogenic signatures (C6 gene sets) were downloaded from the Molecular Signatures Database (http://software.broadinstitute.org/gsea/msigdb/index.jsp, accessed on 6 December 2019). Since microbial data is compositional and to account for the overabundance of zeros in the data, only the reads of microbes with a non-zero read count in more than four of the samples were selected and organized as a continuous variable in the phenotype input. The expression input consisted of human gene expression data in CPM of OA samples. Pearson’s correlation was used to correlate gene and microbe abundance to generate enrichment scores, which reflect the degree of dysregulation in a gene set from a single microbe. High enrichment scores are generated when high microbe abundance is correlated with high gene expression. Using enrichment scores, specific gene sets were ranked based on which ones were being overrepresented. For all statistically significant (*p* < 0.05) associations between canonical pathways and microbes, the -log *p*-value of the microbe-pathway pair was plotted on the heatmap.

### 4.5. Gene Co-Expression Analysis

Only the nine GSEA-correlated microbes were used for this analysis. Patients with lower or higher microbial read counts than the average microbial read count of a particular microbe across all patients were defined as “LOW” or “HIGH,” respectively. The Kruskal–Wallis test was performed by comparing the categorical microbe data to OA-associated/immune-associated gene expression to investigate which significant microbes were significantly associated with significant genes. Only microbe–gene pairs with *p*-values less than 0.05 were deemed significant.

Using Weighted Gene Correlation Network Analysis (WGCNA), the significantly correlated genes were clustered into 20 separate groups, consisting of 10 negative and 10 positive clusters [63]. After reorganization of the significant microbe–gene pairs from Kruskal–Wallis into clusters, the entire cluster of genes were correlated to the GSEA-correlated microbes. Each list of genes from the 20 clusters were then separately analyzed using the Cytoscape ReactomeFIViz plugin to identify the pathways in each cluster. Boxplots were then created correlating the top genes from each pathway to microbes using the Kruskal–Wallis test.

### 4.6. CIBERSORTx

The CIBERSORTx algorithm was used to deconvolute RNA-sequencing data to estimate the infiltration levels of 22 immune cell types [64]. These immune cell types include: naïve B-cells, memory B-cells, plasma cells, CD8 T-cells, CD4 naïve T-cells, CD4 memory resting T-cells, CD4 memory activated T-cells, follicular helper T-cells, regulatory T-cells, gamma-delta T-cells, resting NK cells, activated NK cells, monocytes, M0-M2 macrophages, resting dendritic cells, activated dendritic cells, resting mast cells, activated mast cells, eosinophils, and neutrophils. We then correlated microbe abundance with expression levels of the different immune cells. The Kruskal–Wallis test was employed to correlate immune cell expression levels with microbial abundance.

### 4.7. Contamination Correction Using Read Depth

Scatter plots using Spearman’s correlation were produced, associating individual microbe abundance to total microbe reads in each patient. Normal and OA samples were correlated separately. The scatter plots that exhibited a nearly vertical regression line deemed the microbe a contaminant, but a positive slope with linear association deemed the microbe authentic.

## Supplementary Materials

Supplementary Materials can be found at https://www.mdpi.com/1422-0067/21/22/8618/s1. Figure S1: Remaining GSEA plots from the nine key microbes with the strongest correlated pathway for each microbe not included in Figure 3B. Figure S2: Gene set enrichment analysis (GSEA) correlations of significantly upregulated *Pseudomonas* with (A) canonical pathways and (B) immunologic signatures. (nominal *p* < 0.05). Figure S3: Gene set enrichment analysis (GSEA) correlations of significantly upregulated microbes, including both Unidentified Eubacterium Clone *ESH20b-4* and Obligately Oligotrophic Bacterium *POCPN-83*, Uncultured Bacterium (77133), and *Cupriavidus Necator*, with (A) canonical pathways and (B) immunologic signatures (nominal *p* < 0.05). Each plot is separated based on microbes of similar ancestry. Figure S4: The remaining non-contaminant microbes in both OA and normal samples that did not have significant correlations to signatures included in the GSEA.

## Abbreviations

OA	Osteoarthritis
CPM	Counts per million
GSEA	Gene Set Enrichment Analysis
KEGG	Kyoto Encyclopedia of Genes and Genotype
WGCNA	Weighted Gene Correlation Network Analysis
ECM	Extracellular Matrix
IA	Immune-Associated
