# Supplementary material for: Identification and Characterization of the Intra-Articular Microbiome in the Osteoarthritic Knee
Source: Int J Mol Sci. 2020 Nov 16;21(22):8618. doi: 10.3390/ijms21228618 (PMC7697780; doi:10.3390/ijms21228618)
Supplement: Supplementary file 1 [file ijms-21-08618-s001.zip › SupFig3.pdf]

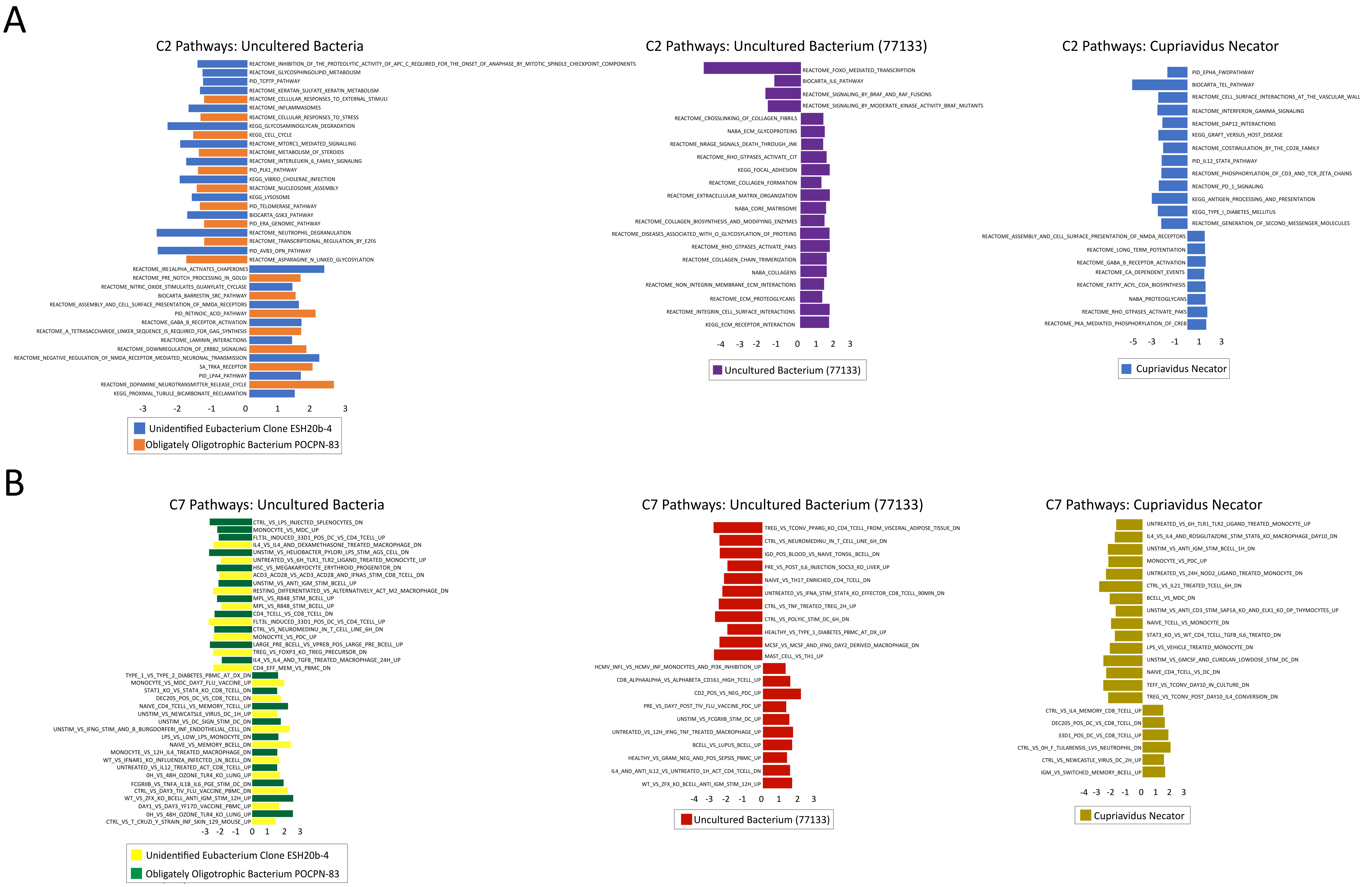
A
C2 Pathways: Uncultered Bacteria
REACTOME_INHIBITION_OF_THE_PROTEOLYTIC_ACTIVITY_OF_APC_C_REQUIRED_FOR_THE_ONSET_OF_ANAPHASE_BY_MITOTIC_SPINDLE_CHECKPOINT_COMPONENTS
REACTOME_GLYCOSPHINGOLIPID_METABOLISM
PID_TCPTP_PATHWAY
REACTOME_KERATAN_SULFATE_KERATIN_METABOLISM
REACTOME_CELLULAR_RESPONSES_TO_EXTERNAL_STIMULI
REACTOME_INFLAMMASOMES
REACTOME_CELLULAR_RESPONSES_TO_STRESS
KEGG_GLYCOSAMINOGLYCAN_DEGRADATION
KEGG_CELL_CYCLE
REACTOME_MTORC1_MEDIATED_SIGNALLING
REACTOME_METABOLISM_OF_STEROIDS
REACTOME_INTERLEUKIN_6_FAMILY_SIGNALING
PID_PLK1_PATHWAY
KEGG_VIBRIO_CHOLERAE_INFECTION
REACTOME_NUCLEOSOME_ASSEMBLY
KEGG_LYSOSOME
PID_TELOMERASE_PATHWAY
BIOCARTA_GSK3_PATHWAY
PID_ERA_GENOMIC_PATHWAY
REACTOME_NEUTROPHIL_DEGRANULATION
REACTOME_TRANSCRIPTIONAL_REGULATION_BY_E2F6
PID_AVB3_OPN_PATHWAY
REACTOME_ASPARAGINE_N_LINKED_GLYCOSYLATION
REACTOME_IRE1ALPHA_ACTIVATES_CHAPERONES
REACTOME_PRE_NOTCH_PROCESSING_IN_GOLGI
REACTOME_NITRIC_OXIDE_STIMULATES_GUANYLATE_CYCLASE
BIOCARTA_BARRESTIN_SRC_PATHWAY
REACTOME_ASSEMBLY_AND_CELL_SURFACE_PRESENTATION_OF_NMDA_RECEPTORS
PID_RETINOIC_ACID_PATHWAY
REACTOME_GABA_B_RECEPTOR_ACTIVATION
REACTOME_A_TETRASACCHARIDE_LINKER_SEQUENCE_IS_REQUIRED_FOR_GAG_SYNTHESIS
REACTOME_LAMININ_INTERACTIONS
REACTOME_DOWNREGULATION_OF_ERBB2_SIGNALING
REACTOME_NEGATIVE_REGULATION_OF_NMDA_RECEPTOR_MEDIATED_NEURONAL_TRANSMISSION
SA_TRKA_RECEPTOR
PID_LPA4_PATHWAY
REACTOME_DOPAMINE_NEUROTRANSMITTER_RELEASE_CYCLE
KEGG_PROXIMAL_TUBULE_BICARBONATE_RECLAMATION
-3 -2 -1 0 1 2 3
Unidentified Eubacterium Clone ESH20b-4
Obligately Oligotrophic Bacterium POCPN-83
C2 Pathways: Uncultured Bacterium (77133)
REACTOME_FOXO_MEDIATED_TRANSCRIPTION
BIOCARTA_IL6_PATHWAY
REACTOME_SIGNALING_BY_BRAF_AND_RAF_FUSIONS
REACTOME_SIGNALING_BY_MODERATE_KINASE_ACTIVITY_BRAF_MUTANTS
REACTOME_CROSSLINKING_OF_COLLAGEN_FIBRILS
NABA_ECM_GLYCOPROTEINS
REACTOME_NRAGE_SIGNALS_DEATH_THROUGH_JNK
REACTOME_RHO_GTPASES_ACTIVATE_CIT
KEGG_FOCAL_ADHESION
REACTOME_COLLAGEN_FORMATION
REACTOME_EXTRACELLULAR_MATRIX_ORGANIZATION
NABA_CORE_MATRISOME
REACTOME_COLLAGEN_BIOSYNTHESIS_AND_MODIFYING_ENZYMES
REACTOME_DISEASES_ASSOCIATED_WITH_O_GLYCOSYLATION_OF_PROTEINS
REACTOME_RHO_GTPASES_ACTIVATE_PAKS
REACTOME_COLLAGEN_CHAIN_TRIMERIZATION
NABA_COLLAGENS
REACTOME_NON_INTEGRIN_MEMBRANE_ECM_INTERACTIONS
REACTOME_ECM_PROTEOGLYCANS
REACTOME_INTEGRIN_CELL_SURFACE_INTERACTIONS
KEGG_ECM_RECEPTOR_INTERACTION
-4 -3 -2 -1 0 1 2 3
Uncultured Bacterium (77133)
C2 Pathways: Cupriavidus Necator
PID_EPHA_FWDPATHWAY
BIOCARTA_TEL_PATHWAY
REACTOME_CELL_SURFACE_INTERACTIONS_AT_THE_VASCULAR_WALL
REACTOME_INTERFERON_GAMMA_SIGNALING
REACTOME_DAP12_INTERACTIONS
KEGG_GRAFT_VERSUS_HOST_DISEASE
REACTOME_COSTIMULATION_BY_THE_CD28_FAMILY
PID_IL12_STAT4_PATHWAY
REACTOME_PHOSPHORYLATION_OF_CD3_AND_TCR_ZETA_CHAINS
REACTOME_PD_1_SIGNALING
KEGG_ANTIGEN_PROCESSING_AND_PRESENTATION
KEGG_TYPE_I_DIABETES_MELLITUS
REACTOME_GENERATION_OF_SECOND_MESSENGER_MOLECULES
REACTOME_ASSEMBLY_AND_CELL_SURFACE_PRESENTATION_OF_NMDA_RECEPTORS
REACTOME_LONG_TERM_POTENTIATION
REACTOME_GABA_B_RECEPTOR_ACTIVATION
REACTOME_CA_DEPENDENT_EVENTS
REACTOME_FATTY_ACYL_COA_BIOSYNTHESIS
NABA_PROTEOGLYCANS
REACTOME_RHO_GTPASES_ACTIVATE_PAKS
REACTOME_PKA_MEDIATED_PHOSPHORYLATION_OF_CREB
-5 -3 -1 1 3
Cupriavidus Necator
B
C7 Pathways: Uncultured Bacteria
CTRL_VS_LPS_INJECTED_SPLENOCYTES_DN
MONOCYTE_VS_MDC_UP
FLT3L_INDUCED_33D1_POS_DC_VS_CD4_TCELL_UP
IL4_VS_IL4_AND_DEXAMETHASONE_TREATED_MACROPHAGE_DN
UNSTIM_VS_HELIOBACTER_PYLORI_LPS_STIM_AGS_CELL_DN
UNTREATED_VS_6H_TLR1_TLR2_LIGAND_TREATED_MONOCYTE_UP
HSC_VS_MEGAKARYOCYTE_ERYTHROID_PROGENITOR_DN
ACD3_ACD28_VS_ACD3_ACD28_AND_IFNA5_STIM_CD8_TCELL_DN
UNSTIM_VS_ANTI_IGM_STIM_BCELL_UP
RESTING_DIFFERENTIATED_VS_ALTERNATIVELY_ACT_M2_MACROPHAGE_DN
MPL_VS_R848_STIM_BCELL_UP
MPL_VS_R848_STIM_BCELL_UP
CD4_TCELL_VS_CD8_TCELL_DN
FLT3L_INDUCED_33D1_POS_DC_VS_CD4_TCELL_UP
CTRL_VS_NEUROMEDINU_IN_T_CELL_LINE_6H_DN
MONOCYTE_VS_PDC_UP
LARGE_PRE_BCELL_VS_VPREB_POS_LARGE_PRE_BCELL_UP
TREG_VS_FOXP3_KO_TREG_PRECURSOR_DN
IL4_VS_IL4_AND_TGFB_TREATED_MACROPHAGE_24H_UP
CD4_EFF_MEM_VS_PBMC_DN
TYPE_1_VS_TYPE_2_DIABETES_PBMC_AT_DX_DN
MONOCYTE_VS_MDC_DAY7_FLU_VACCINE_UP
STAT1_KO_VS_STAT4_KO_CD8_TCELL_DN
DEC205_POS_DC_VS_CD8_TCELL_DN
NAIVE_CD4_TCELL_VS_MEMORY_TCELL_UP
UNSTIM_VS_NEWCATSLE_VIRUS_DC_1H_UP
UNSTIM_VS_DC_SIGN_STIM_DC_DN
UNSTIM_VS_IFNG_STIM_AND_B_BURGDORFERI_INF_ENDOTHELIAL_CELL_DN
LPS_VS_LOW_LPS_MONOCYTE_DN
NAIVE_VS_MEMORY_BCELL_DN
MONOCYTE_VS_12H_IL4_TREATED_MACROPHAGE_DN
WT_VS_IFNAR1_KO_INFLUENZA_INFECTED_LN_BCELL_DN
UNTREATED_VS_IL12_TREATED_ACT_CD8_TCELL_UP
0H_VS_48H_OZONE_TLR4_KO_LUNG_UP
FCGRIIB_VS_TNFA_IL1B_IL6_PGE_STIM_DC_DN
CTRL_VS_DAY3_TIV_FLU_VACCINE_PBMC_DN
WT_VS_ZFX_KO_BCELL_ANTI_IGM_STIM_12H_UP
DAY1_VS_DAY3_YF17D_VACCINE_PBMC_UP
0H_VS_48H_OZONE_TLR4_KO_LUNG_UP
CTRL_VS_T_CRUZI_Y_STRAIN_INF_SKIN_129_MOUSE_UP
-3 -2 -1 0 1 2 3
Unidentified Eubacterium Clone ESH20b-4
Obligately Oligotrophic Bacterium POCPN-83
C7 Pathways: Uncultured Bacterium (77133)
TREG_VS_TCONV_PPARG_KO_CD4_TCELL_FROM_VISCERAL_ADIPOSE_TISSUE_DN
CTRL_VS_NEUROMEDINU_IN_T_CELL_LINE_6H_DN
IGD_POS_BLOOD_VS_NAIVE_TONSIL_BCELL_DN
PRE_VS_POST_IL6_INJECTION_SOCS3_KO_LIVER_UP
NAIVE_VS_TH17_ENRICHED_CD4_TCELL_DN
UNTREATED_VS_IFNA_STIM_STAT4_KO_EFFECTOR_CD8_TCELL_90MIN_DN
CTRL_VS_TNF_TREATED_TREG_2H_UP
CTRL_VS_POLYIC_STIM_DC_6H_DN
HEALTHY_VS_TYPE_1_DIABETES_PBMC_AT_DX_UP
MCSF_VS_MCSF_AND_IFNG_DAY2_DERIVED_MACROPHAGE_DN
MAST_CELL_VS_TH1_UP
HCMV_INFL_VS_HCMV_INF_MONOCYTES_AND_PI3K_INHIBITION_UP
CD8_ALPHAALPHA_VS_ALPHABETA_CD161_HIGH_TCELL_UP
CD2_POS_VS_NEG_PDC_UP
PRE_VS_DAY7_POST_TIV_FLU_VACCINE_PDC_UP
UNSTIM_VS_FCGRIIB_STIM_DC_UP
UNTREATED_VS_12H_IFNG_TNF_TREATED_MACROPHAGE_UP
BCELL_VS_LUPUS_BCELL_UP
HEALTHY_VS_GRAM_NEG_AND_POS_SEPSIS_PBMC_UP
IL4_AND_ANTI_IL12_VS_UNTREATED_1H_ACT_CD4_TCELL_DN
WT_VS_ZFX_KO_BCELL_ANTI_IGM_STIM_12H_UP
-4 -3 -2 -1 0 1 2 3
Uncultured Bacterium (77133)
C7 Pathways: Cupriavidus Necator
UNTREATED_VS_6H_TLR1_TLR2_LIGAND_TREATED_MONOCYTE_UP
IL4_VS_IL4_AND_ROSIGLITAZONE_STIM_STAT6_KO_MACROPHAGE_DAY10_DN
UNSTIM_VS_ANTI_IGM_STIM_BCELL_1H_DN
MONOCYTE_VS_PDC_UP
UNTREATED_VS_24H_NOD2_LIGAND_TREATED_MONOCYTE_DN
CTRL_VS_IL21_TREATED_TCELL_6H_DN
BCELL_VS_MDC_DN
UNSTIM_VS_ANTI_CD3_STIM_SAP1A_KO_AND_ELK1_KO_DP_THYMOCYTES_UP
NAIVE_TCELL_VS_MONOCYTE_DN
STAT3_KO_VS_WT_CD4_TCELL_TGFB_IL6_TREATED_DN
LPS_VS_VEHICLE_TREATED_MONOCYTE_DN
UNSTIM_VS_GMCSF_AND_CURDLAN_LOWDOSE_STIM_DC_DN
NAIVE_CD4_TCELL_VS_DC_DN
TEFF_VS_TCONV_DAY10_IN_CULTURE_DN
TREG_VS_TCONV_POST_DAY10_IL4_CONVERSION_DN
CTRL_VS_IL4_MEMORY_CD8_TCELL_UP
DEC205_POS_DC_VS_CD8_TCELL_DN
33D1_POS_DC_VS_CD8_TCELL_UP
CTRL_VS_0H_F_TULARENSIS_LVS_NEUTROPHIL_DN
CTRL_VS_NEWCASTLE_VIRUS_DC_2H_UP
IGM_VS_SWITCHED_MEMORY_BCELL_UP
-4 -3 -2 -1 0 1 2 3
Cupriavidus Necator
