# Supplementary material for: Identification and Characterization of the Intra-Articular Microbiome in the Osteoarthritic Knee
Source: Int J Mol Sci. 2020 Nov 16;21(22):8618. doi: 10.3390/ijms21228618 (PMC7697780; doi:10.3390/ijms21228618)

Pseudomonas Azotoformans

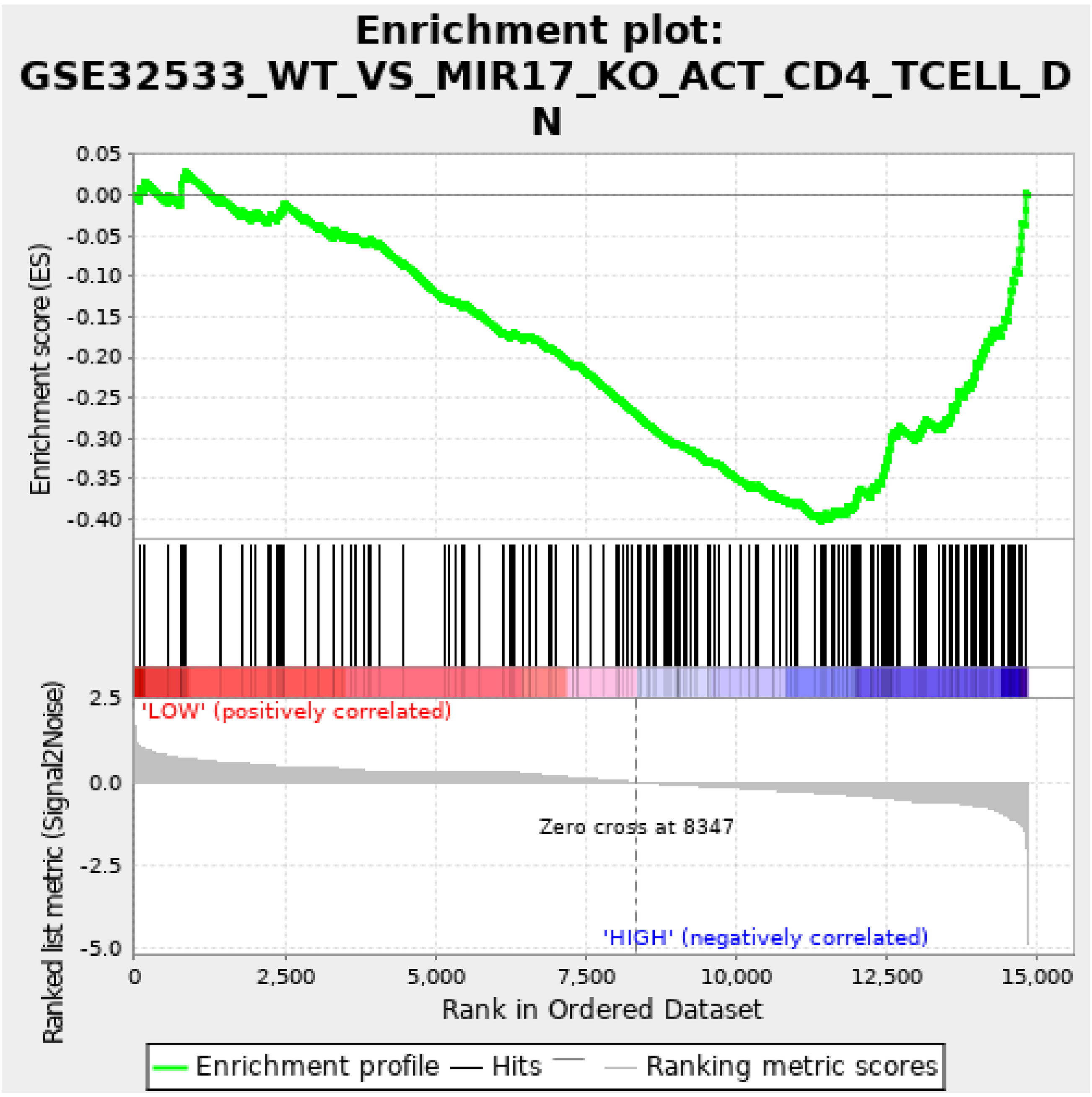

Obligately Oligotrophic Bacterium POCPN-83

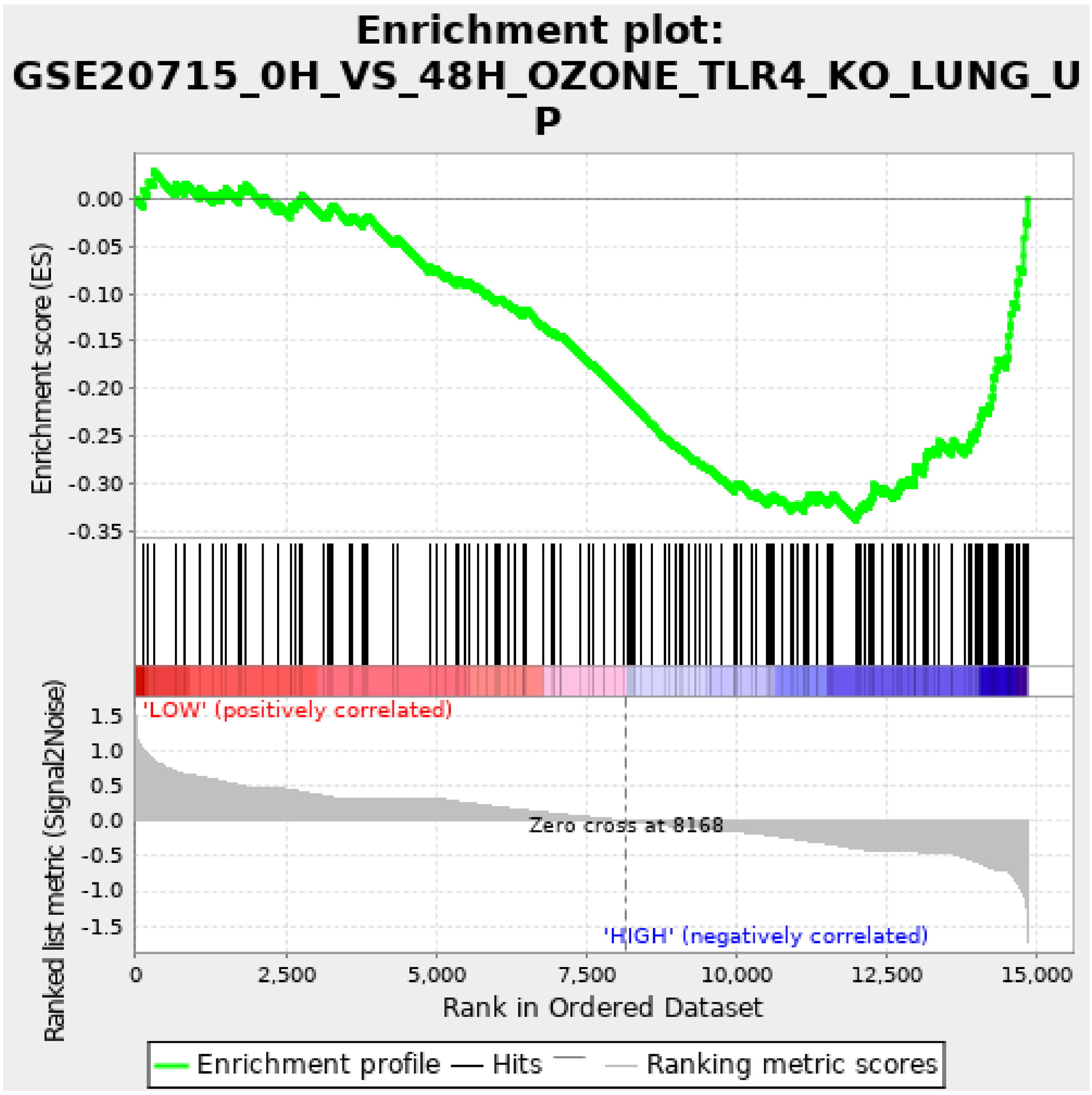

Pseudomonas Migulae

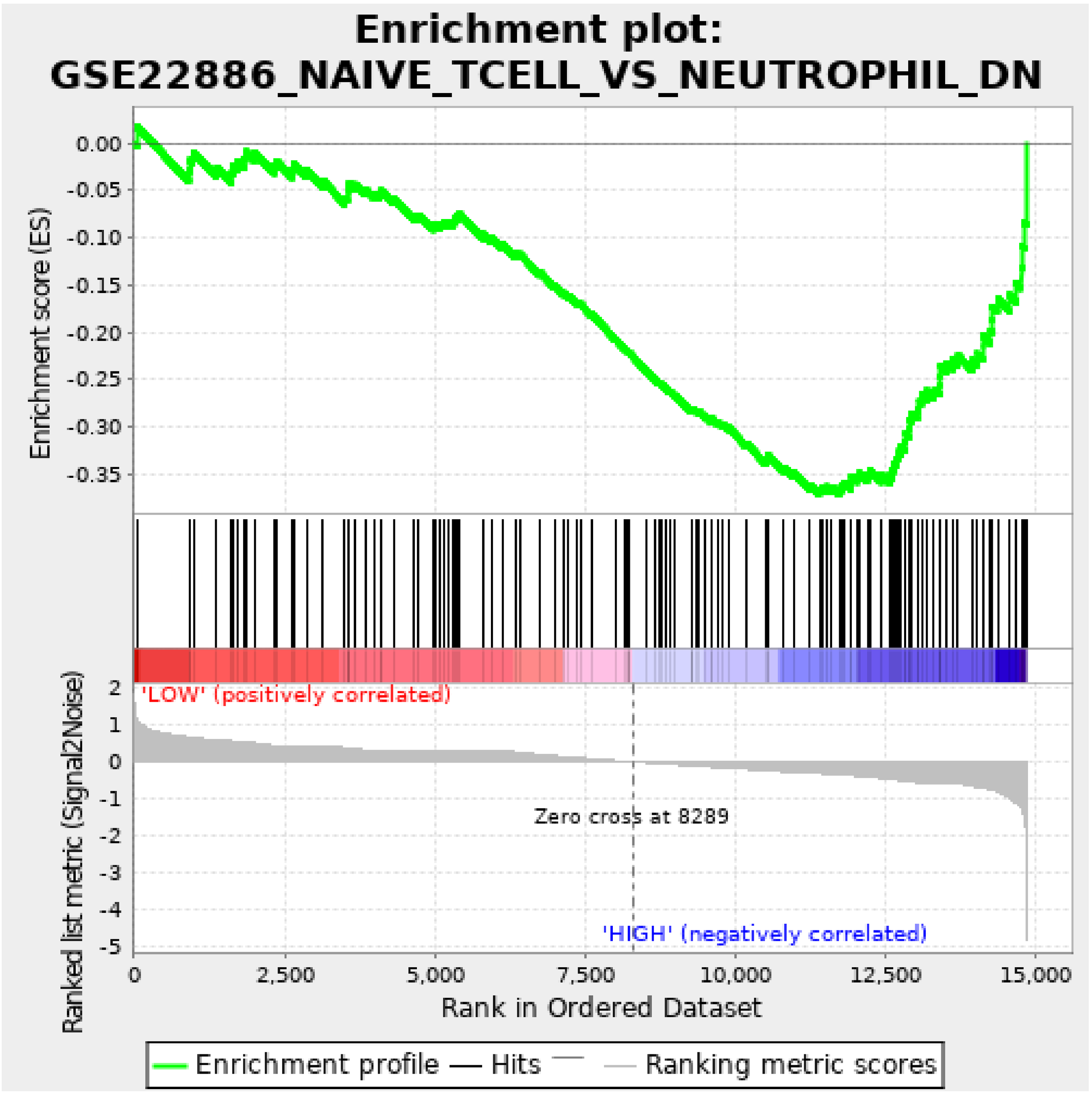

Unidentified Eubacterium Clone ESH20b-4

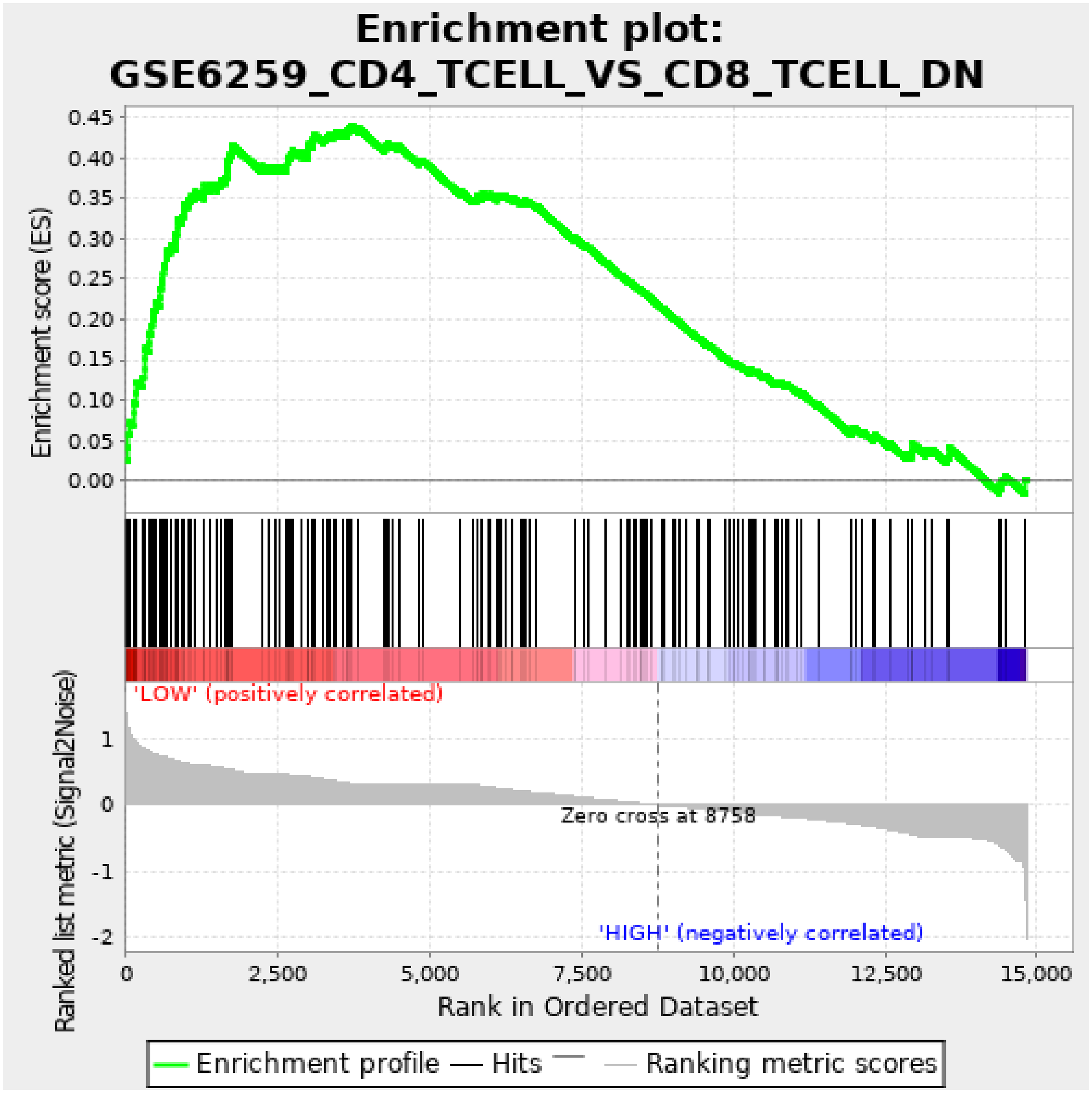

Uncultered Bacterium (77133)

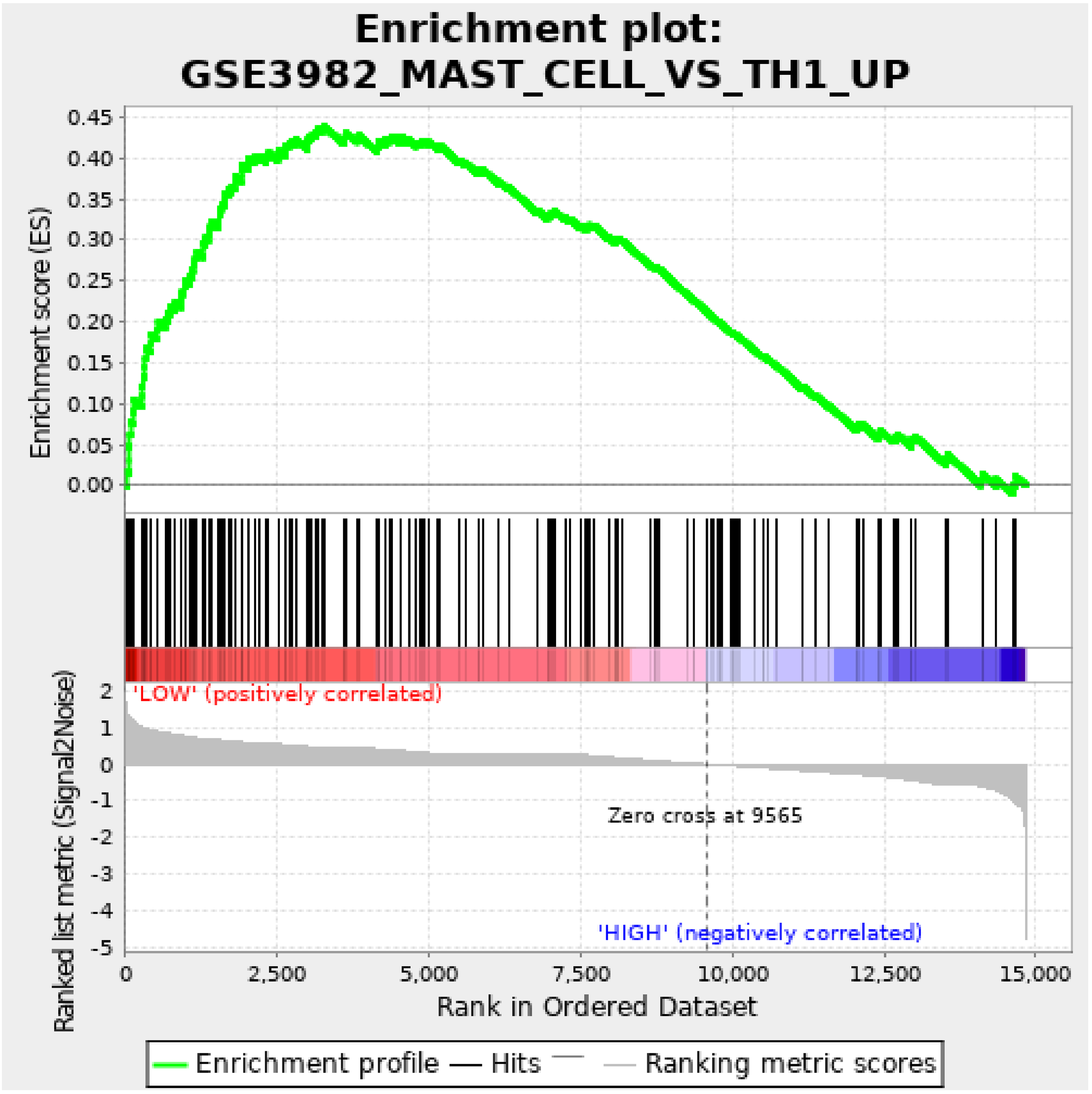

Cupriavidus Necator

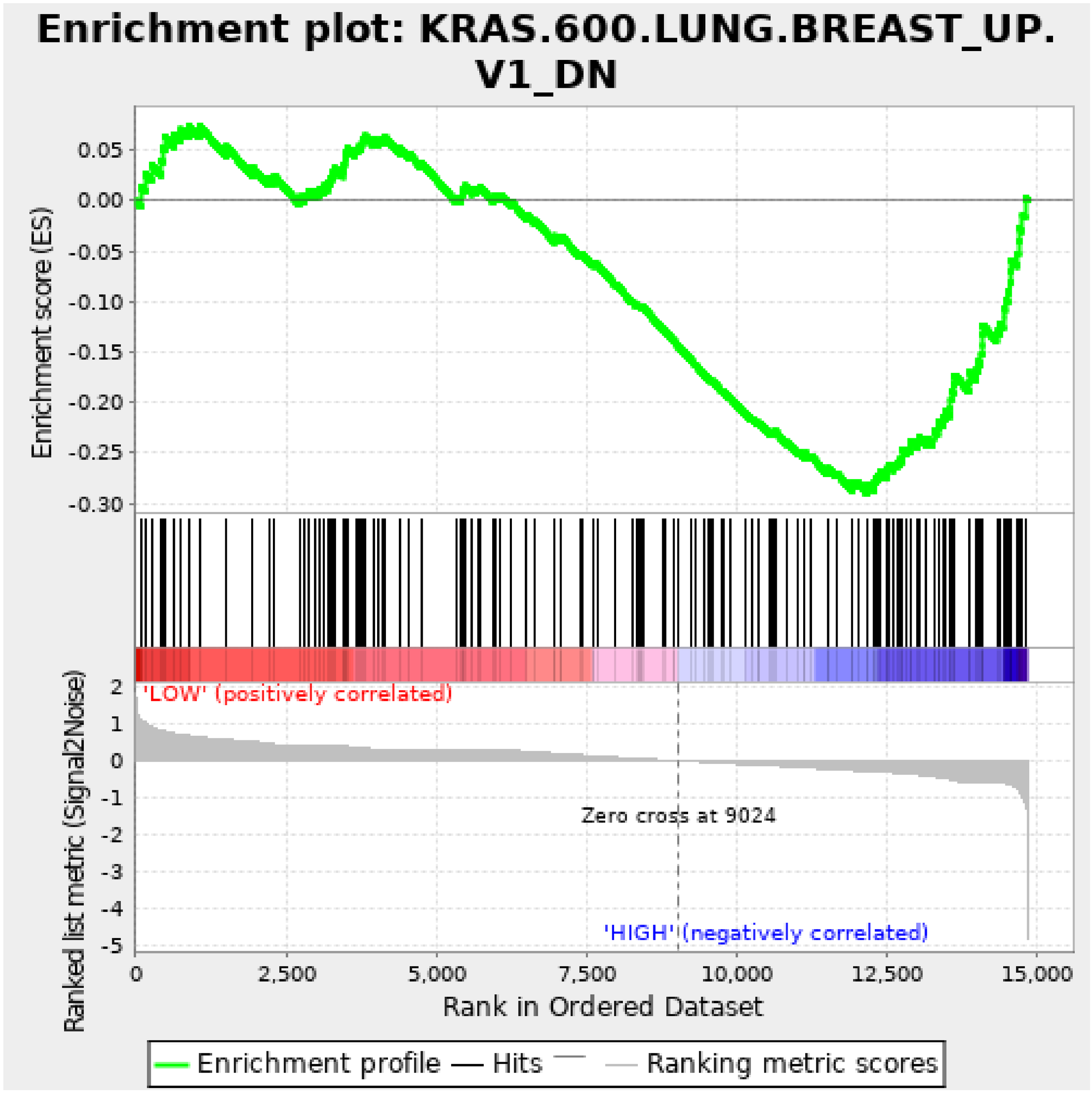

Supplement: Supplementary file 1 [file ijms-21-08618-s001.zip › SupFig1.pdf]
