# Supplementary figures and images for: Identification and Characterization of the Intra-Articular Microbiome in the Osteoarthritic Knee
Source: Int J Mol Sci. 2020 Nov 16;21(22):8618. doi: 10.3390/ijms21228618 (PMC7697780; doi:10.3390/ijms21228618)

# Normals

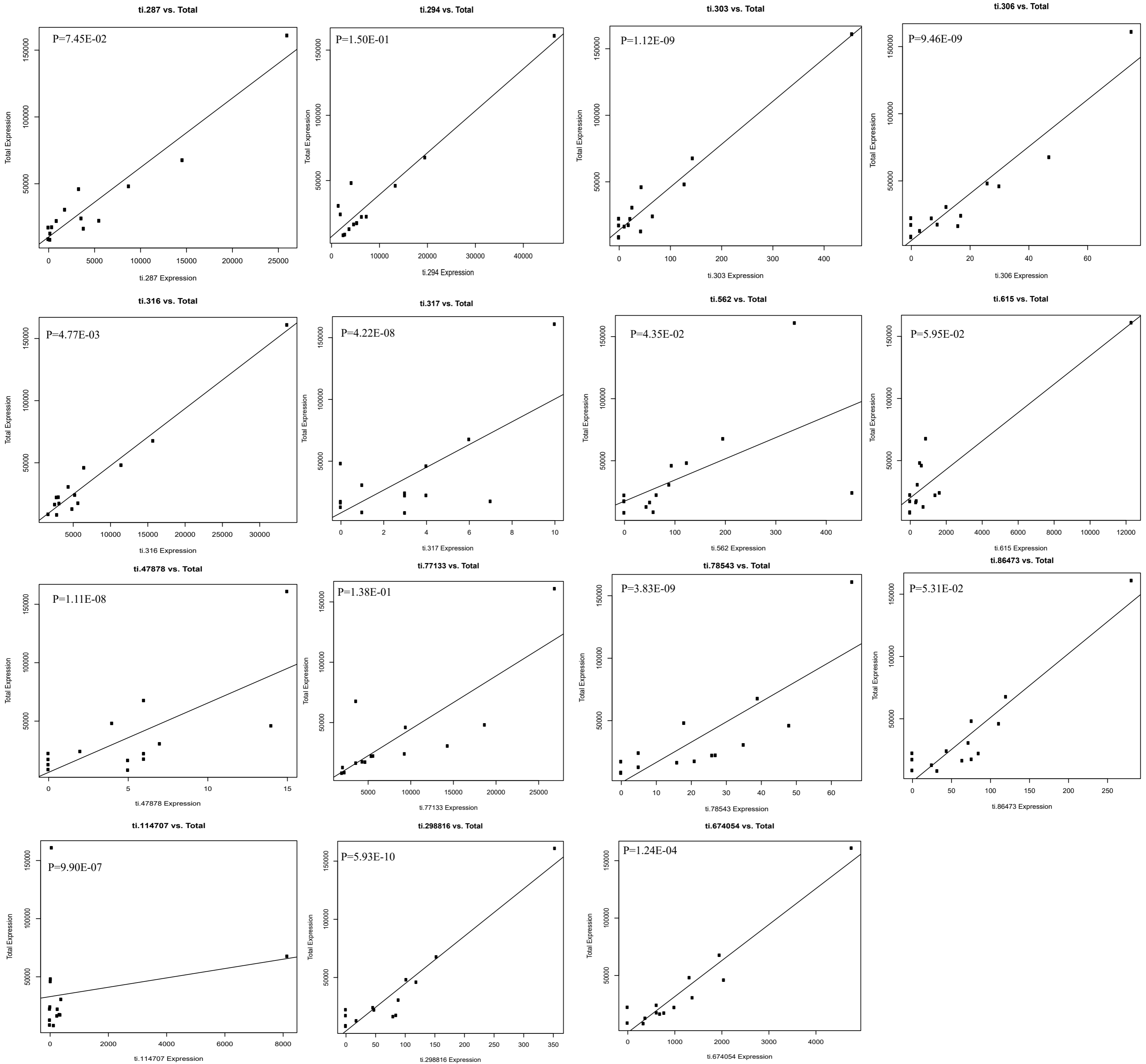

# Diseased

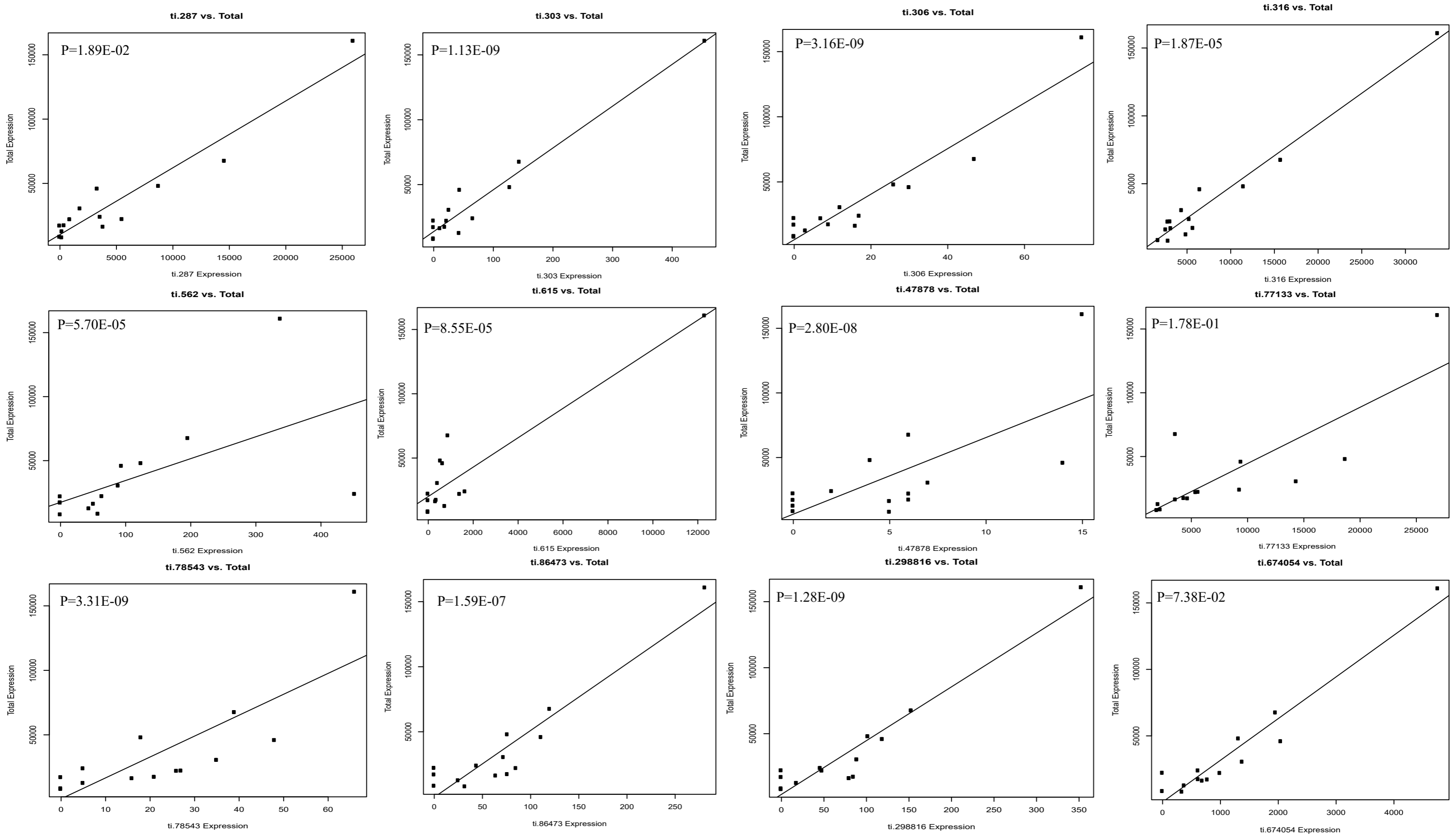

Supplement: Supplementary file 1 [file ijms-21-08618-s001.zip › SupFig4.pdf]

A

C2 Pathways: Pseudomonas

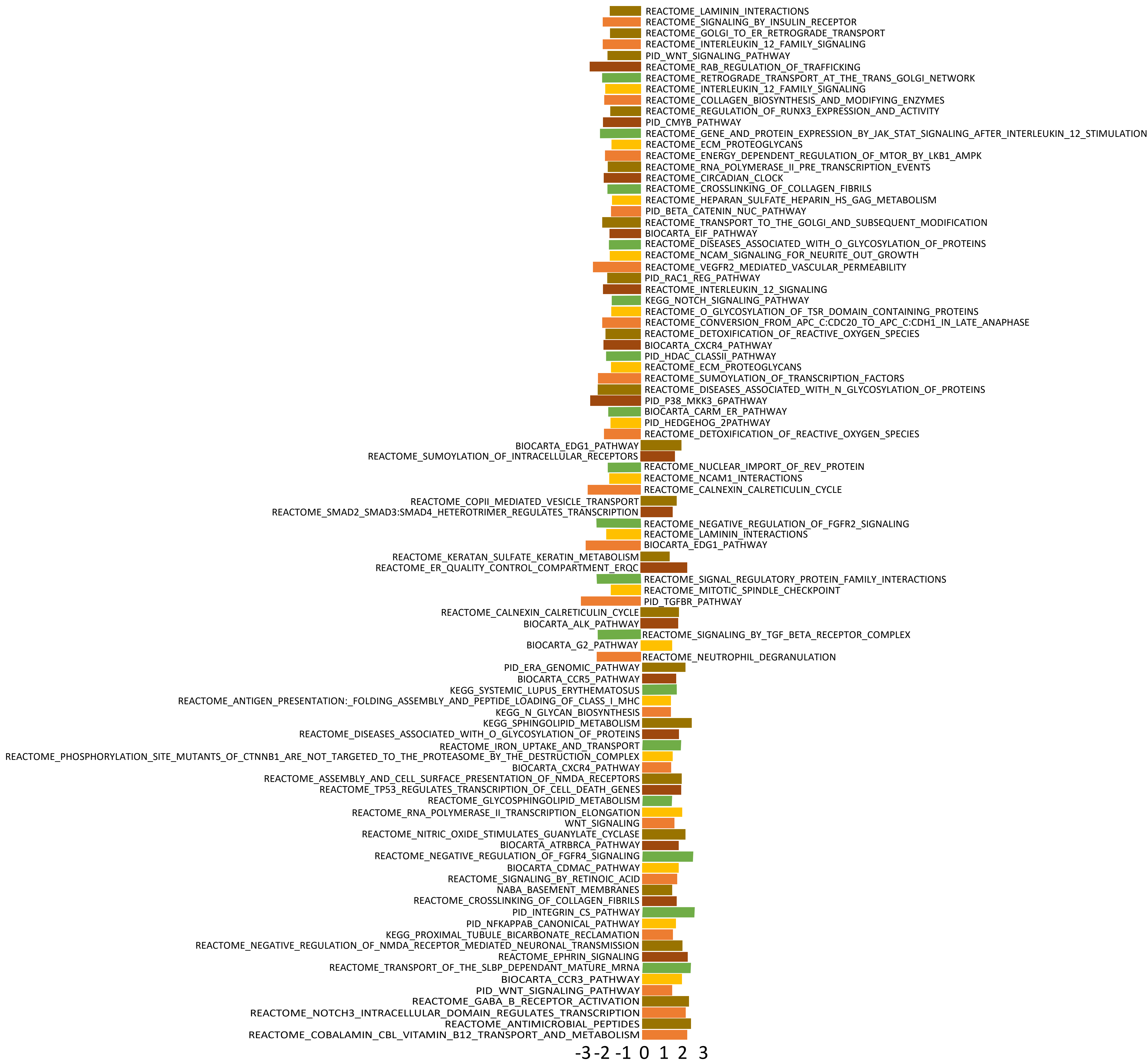

B

C7 Pathways: Pseudomonas

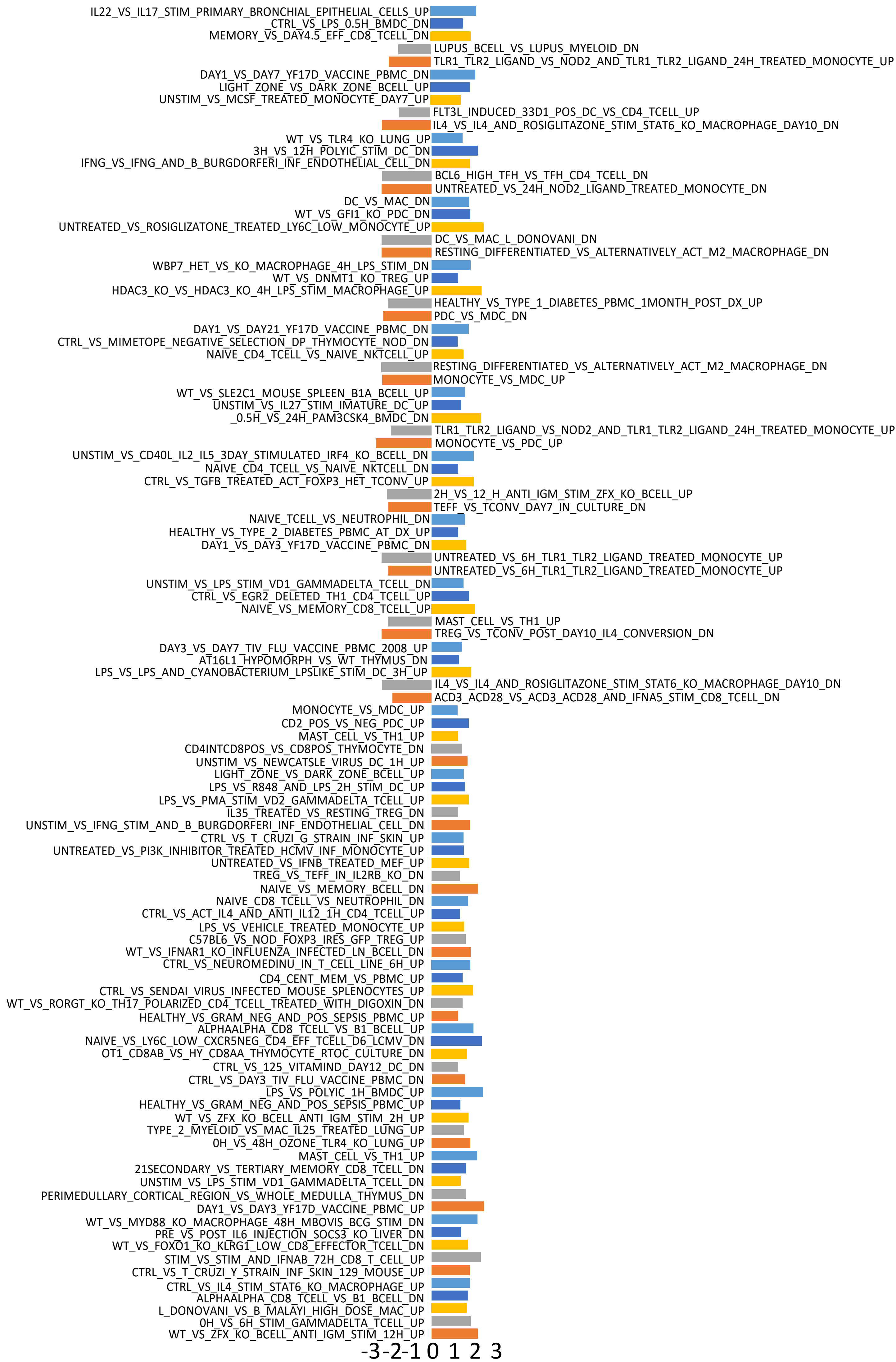

Supplement: Supplementary file 1 [file ijms-21-08618-s001.zip › SupFig2.pdf]
